# Hepatic rupture complicating HELLP syndrome in a low-resource setting: a case report

**DOI:** 10.1186/s12884-026-09175-1

**Published:** 2026-04-30

**Authors:** Mihiretu Tesfamariam Goshu, Ephrem Kedir

**Affiliations:** Department of Obstetrics and Gynecology, School of Medicine and Health Sciences, Hawassa University, Hawassa, Ethiopia

**Keywords:** HELLP syndrome, Hepatic rupture, Preeclampsia, Hemoperitoneum, Subcapsular hematoma

## Abstract

Spontaneous hepatic rupture is a rare but life-threatening complication of Hemolysis, elevated liver enzymes, and low platelet count (HELLP) syndrome, associated with high maternal and perinatal mortality. Its diagnosis is challenging due to a nonspecific clinical presentation and limited diagnostic resources in low-resource settings. We report a 35-year-old woman who claims 7 months of amenorrhea with the diagnosis of severe preeclampsia and HELLP syndrome, who developed sudden right upper quadrant pain and hemodynamic instability. The bedside ultrasound showed hemoperitoneum and a ruptured subcapsular hematoma. Emergency laparotomy confirmed hepatic rupture, and hemostasis was achieved with surgical intervention. This case highlights the importance of maintaining a high index of suspicion for hepatic rupture in patients with HELLP syndrome presenting with acute abdominal pain. Timely diagnosis and surgical management are critical to improving maternal outcomes.

## Introduction

Spontaneous hepatic rupture is a rare but life-threatening complication of HELLP syndrome, which is characterized by microangiopathic hemolysis, low platelet count, and elevated liver enzymes [1]. HELLP syndrome occurs in approximately 10–20% of women with severe preeclampsia [2]. Hepatic complications such as hepatic hematoma and hepatic rupture occur in 1 to 2% of HELLP syndrome and are associated with a maternal mortality rate of up to 17 to 59% [3, 4]. The clinical presentation is often nonspecific, including right upper quadrant pain, hypotension, and signs of hypovolemic shock.

The best diagnostic modality is a contrast-enhanced CT scan of the abdomen in hemodynamically stable patients, whereas bedside ultrasound is recommended for unstable patients, although in some cases hepatic rupture may be diagnosed unexpectedly during emergency laparotomy. In hemodynamically unstable patients, immediate surgical intervention is necessary, including emergency laparotomy with perihepatic packing, hepatic artery ligation, partial hepatectomy, liver transplantation, and massive transfusion. In contrast, hemodynamically stable patients with a contained subcapsular hematoma may be managed conservatively [5]. We report a patient who presented with hemoperitoneum due to liver rupture secondary to HELLP syndrome and managed surgically in a low-resource setting.

## Case report

A 35-year-old gravida III para II woman who claims 7 months of amenorrhea presented with persistent headache, blurring of vision, and mild epigastric pain for a four-day duration. She also reported tinnitus, vertigo, easy fatigability, two episodes of moderate vaginal bleeding, and generalized body swelling for two weeks. There was no history of trauma.

On examination, her blood pressure was 150/95 mmHg, and pulse rate was 120 beats per minute. She had pale conjunctiva, bilateral pitting edema, a 28-week-size gravid uterus, a positive fetal heartbeat, and clinical signs of an intra-abdominal fluid collection. Upon admission, her laboratory results showed a hematocrit of 18%, a platelet count of 48,000/µL, ALT of 54 U/L, AST of 39 U/L, and a serum creatinine of 0.7 mg/dL.

A diagnosis of severe preeclampsia with HELLP syndrome was made. Magnesium sulphate was initiated, and she was transfused with one unit of packed red blood cells. Four hours after admission, she developed sudden severe epigastric and right upper quadrant pain associated with pleuritic chest pain, and inability to lie supine. Her blood pressure decreased to 110/70 mmHg, and her pulse increased to 130 beats per minute. Abdominal examination revealed diffuse epigastric and right upper quadrant tenderness with increasing abdominal girth. The fetal heartbeat became negative.

**Fig. 1:**
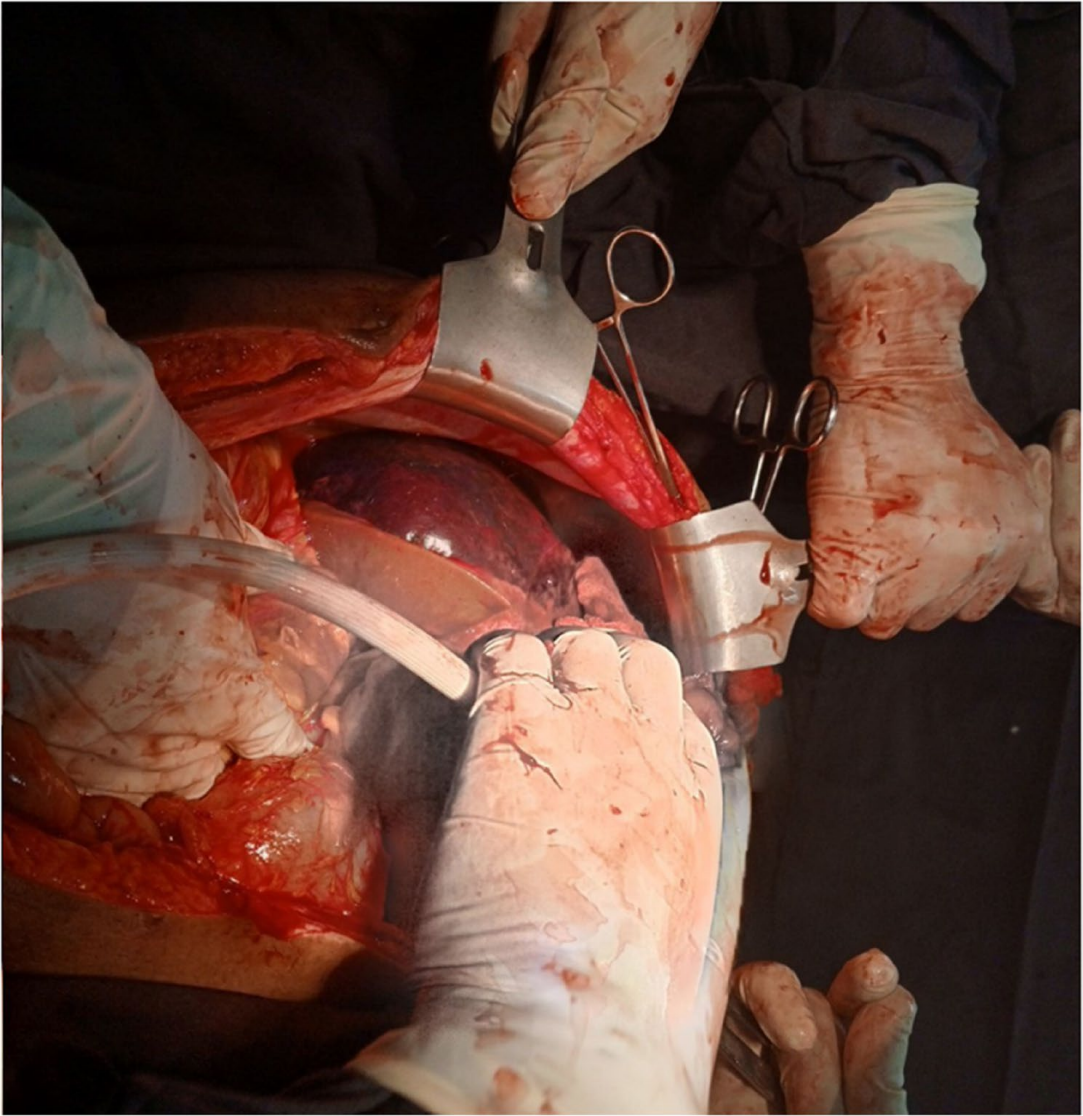
Intraoperative image showing a large subcapsular hepatic hematoma involving approximately 50% of the liver surface and elevating the liver capsule

**Fig. 2:**
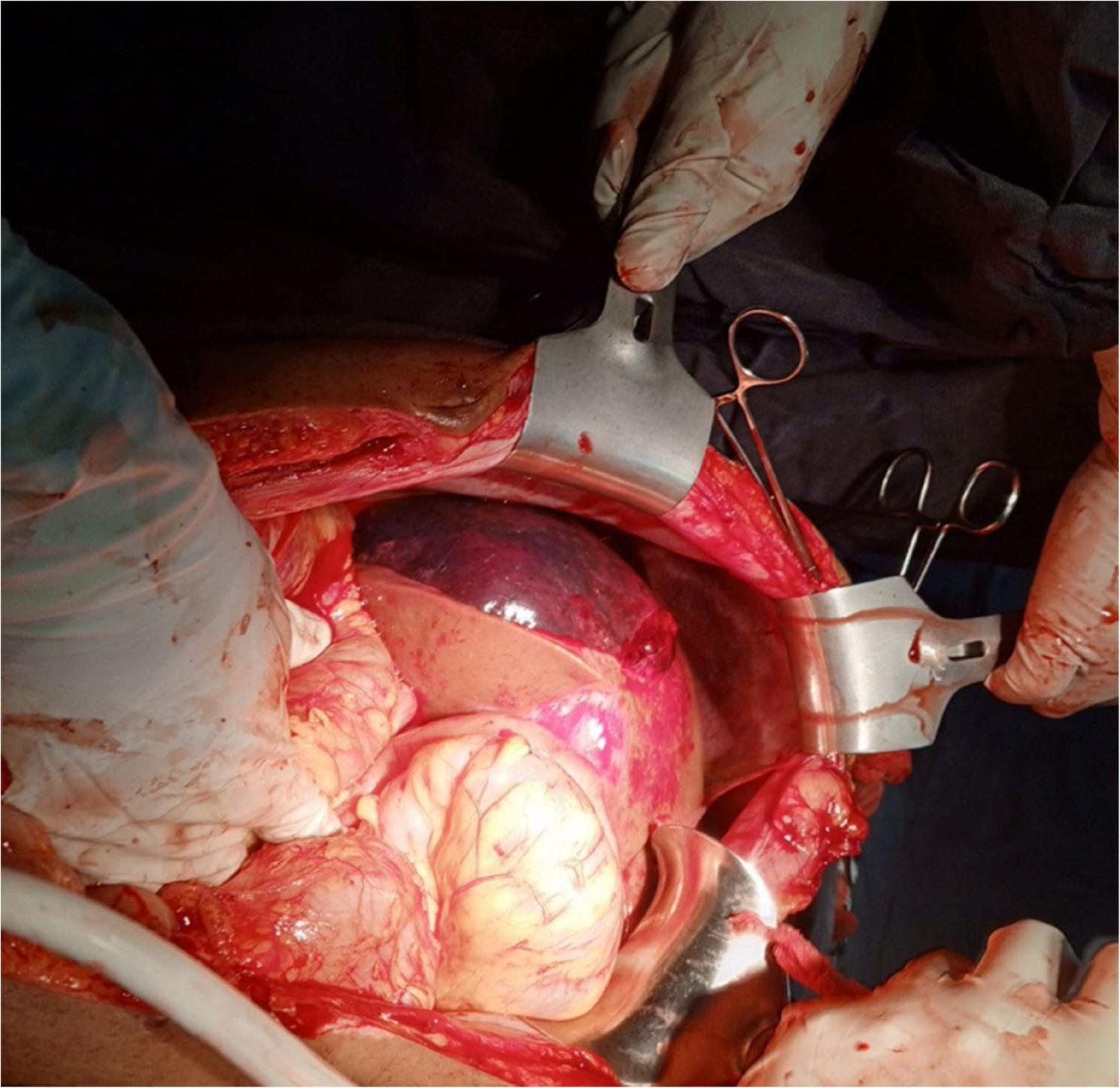
Intraoperative image showing rupture of a subcapsular hepatic hematoma at the right subdiaphragmatic region with hemoperitoneum and a disrupted liver capsule

Repeat investigation showed that worsening anemia (HCT 16%), thrombocytopenia (35,000/µL), elevated liver enzymes (AST 209 U/L, ALT 110 U/L), total bilirubin 2.09 mg/dL (direct 1.33 mg/dL), and serum creatinine 1.3 mg/dL. Free intraperitoneal fluid with internal echoes and a hyperechoic subhepatic lesion with discontinuity of the liver capsule, suggestive of ruptured hepatic hematoma, was seen on bedside ultrasound. With a diagnosis of hepatic rupture secondary to HELLP syndrome, emergency laparotomy was performed. Intraoperatively, 800 ml of hemoperitoneum was sucked out, and lower uterine segment cesarean delivery was performed, delivering a 1.3 kg freshly dead fetus. Inspection of the liver revealed a large subcapsular hematoma involving approximately 50% of the hepatic surface area with rupture at the right subdiaphragmatic region **(**Figs. 1 and 2**)**. Haemostasis was secured using omentopexy over the ruptured area, and an intra-abdominal drain was placed. The patient received a total of six units of blood transfusion and was admitted to the intensive care unit for postoperative monitoring. The patient showed progressive clinical improvement. Drain output gradually decreased, laboratory parameters normalized, and she was discharged in stable condition after full recovery **(**Table 1**).**

**Table 1:** Serial postoperative day investigations

Postoperative Day	ALT (U/L)	AST (U/L)	HCT (%)	Creatinine (mg/dL)
Day 1	762.4	650	29.6	1.19
Day 2	491.8	190.4	27.8	0.90
Day 3	375.8	176.5	25.5	0.70
Day 4	210.7	75	26.2	0.59
Day 5	54.3	41.5	26.3	0.54

Reference ranges: ALT (7–56 U/L), AST (10–40 U/L), HCT (36–46%), creatinine (0.6–1.1 mg/dL)

## Discussion

Hepatic rupture is a life-threatening complication of HELLP syndrome. Patient presents with sudden epigastric or right upper quadrant pain and signs of internal bleeding. Because symptoms initially look similar to worsening preeclampsia, a high index of suspicion is required [6]. Diagnosis is confirmed by imaging studies such as ultrasound and CT scan. Interventional radiological procedures like angiography are useful for both diagnosis and therapy [7]. In our patient, hepatic rupture was diagnosed by bedside ultrasound, which shows free intraperitoneal fluid with internal echoes, a hyperechoic lesion beneath the liver capsule, and discontinuity of the liver capsule.

Hepatic rupture outcomes vary significantly between high-resource and low-resource settings. Maternal survival is better in well-equipped facilities due to early diagnosis with contrast-enhanced CT scan and accessibility to interventional radiology procedures. In contrast, in low-resource settings, limited access to advanced imaging modalities often necessitates reliance on bedside ultrasound. Although ultrasound is fast and easily available, it is operator-dependent and less sensitive in detecting hepatic lesions, particularly in early or contained hematomas [4].

The pathophysiology of hepatic rupture in HELLP syndrome is due to the vasospasm from increased sensitivity to circulating vasopressors during pregnancy, and vascular injury from endothelial damage leads to the formation of microvascular thrombi, which finally result in rupture [8].

Management depends on patient hemodynamic stability. Conservative treatment with serial haemoglobin monitoring, ultrasound, or CT is required. In unstable patients, emergency laparotomy, perihepatic packing, or liver resection can be done [5]. In our case, rapid deterioration and hemodynamic instability necessitated emergency laparotomy and intraoperative omental packing. Although maternal mortality associated with hepatic rupture in HELLP syndrome ranges from 17% to 59%, early multidisciplinary collaboration, adequate blood product replacement, and intensive postoperative care can improve maternal survival [3].

## Conclusion

Spontaneous hepatic rupture is a rare but life-threatening complication of HELLP syndrome. In low-resource settings, clinicians should maintain a high index of suspicion in patients with preeclampsia who develop sudden, severe epigastric or right upper quadrant pain with hemodynamic instability. Early use of bedside ultrasound and timely surgical intervention are essential to reduce maternal mortality.

## Data Availability

No datasets were generated or analysed during the current study.

## Abbreviations

ALT: Alanine Transaminase
AST: Aspartate Aminotransferase
HELLP: Hemolysis, Elevated liver enzymes, and Low platelet count
